# Prenatal paracetamol exposure and neurodevelopmental outcomes in preschool-aged children

**DOI:** 10.1111/ppe.12568

**Published:** 2019-08-25

**Authors:** Johanne N. Trønnes, Mollie Wood, Angela Lupattelli, Eivind Ystrom, Hedvig Nordeng

**Affiliations:** 1 PharmacoEpidemiology and Drug Safety Research Group, Department of Pharmacy, and PharmaTox Strategic Initiative, Faculty of Mathematics and Natural Sciences, University of Oslo, Oslo, Norway; 2 Department of Epidemiology, Harvard TH Chan School of Public Health, Boston, MA, USA; 3 Department of Mental Disorders, Norwegian Institute of Public Health, Oslo, Norway; 4 Department of Psychology, University of Oslo, Oslo, Norway; 5 Department of Child Health and Development, Norwegian Institute of Public Health, Oslo, Norway

**Keywords:** child neurodevelopment, MoBa, paracetamol, pregnancy

## Abstract

**Background:**

Recent studies have suggested an association between prenatal paracetamol exposure and adverse neurodevelopmental outcomes in children. However, these findings may be confounded by unmeasured factors related to maternal use of paracetamol and child outcomes.

**Objective:**

To examine the association between duration and timing of prenatal paracetamol exposure on parent-reported communication skills, behaviour, and temperament in preschool-aged children, with focus on the role of unmeasured confounding.

**Methods:**

We used data from the Norwegian Mother and Child Cohort Study. Linear and generalised linear models with inverse probability weights and robust standard errors were used to quantify the association between prenatal paracetamol exposure and continuous and categorical outcomes.

**Results:**

Of the 32 934 children included in our study, 8374 (25.4%), 4961 (15.1%), and 1791 (5.4%) were prenatally exposed to paracetamol in one, two, and three trimesters, respectively. Children exposed to paracetamol in two trimesters scored lower on shyness compared with unexposed children (β −0.62, 95% confidence interval [CI] −1.05, −0.19). Children exposed to paracetamol in three trimesters had a moderate increased risk of internalising behaviour problems (relative risk (RR) 1.36, 95% CI 1.02, 1.80) and borderline externalising behaviour problems (RR 1.22, 95% CI 0.93, 1.60) compared with unexposed children. Children exposed to paracetamol in 2nd/3rd trimester scored lower on shyness (β −0.32, 95% CI −0.66, 0.02) compared with unexposed children. Sensitivity analyses indicated that unmeasured confounders play an important role and may potentially bias the effect estimates away from the null.

**Conclusions:**

Timing of exposure and short-term use of paracetamol during pregnancy do not seem to pose any substantial risk of the outcomes examined. Although we found an association between paracetamol use in multiple trimesters and lower shyness and greater internalising behaviour in preschool-aged children, we cannot rule out chance or unmeasured confounding as possible explanations for these findings.

## 1 | BACKGROUND

Since 2013, several studies of multiple birth cohorts have suggested an association between paracetamol exposure during pregnancy and adverse neurodevelopmental outcomes in children.^1–9^ Paracetamol crosses the placenta and the blood-brain barrier, and several biologically plausible mechanisms for interfering with foetal brain development have been suggested, including neurotoxicity induced by oxidative stress,^10,11^ interaction with maternal hormones (thyroid and sex hormones) important for normal brain development,^12^ and stimulation of endocannabinoid receptors required for normal axonal growth and fasciculation. However, prior findings may be confounded by unmeasured factors related to maternal use of paracetamol and child outcomes. Given the widespread use of paracetamol among 40%–65% of pregnant women,^13,14^ establishing its long-term neurodevelopmental safety continues to be of great public health interest.

Determining the effect of prenatal paracetamol exposure on child neurodevelopment is challenging. The term “neurodevelopment” encompasses a wide range of domains,^15^ and though previous studies have focused mainly on attention deficit hyperactivity disorder (ADHD) and behavioural outcomes,^9,16^ other outcomes, such as communication skills and temperament, are also important domains within the realm of neurodevelopment. Moreover, bias and confounding are problems encountered with observational data.^17^ In particular, unmeasured confounding poses important challenges, as we do not know the magnitude or direction of bias and cannot account for it fully.^18^ To address unmeasured confounding, two recent studies used paternal paracetamol use as a negative control in relation to child outcomes, with conflicting results.^4,9^ Prior to those two studies, Brandlistuen and colleagues^1^ employed a sibling design, which partially accounts for familial and genetic confounding, and found that long-term paracetamol exposure was associated with adverse neurodevelopmental outcomes in 3-year-old children in the Norwegian Mother and Child Cohort Study.

It is important to examine the association between paracetamol use in pregnancy and child neurodevelopment at different child ages.^19^ We build on previous research within the Norwegian Mother and Child Cohort Study (MoBa)^1,8^ and reassesses child neurodevelopment at 5 years. We investigate the association between prenatal exposure to paracetamol and communication, externalising and internalising behaviour, and temperament in preschool-aged children and explore the role of unmeasured confounding.

## 2 | METHODS

### 2.1 | Study population and data collection

This is a sub-study of the MoBa conducted by the Norwegian Institute of Public Health. The MoBa is a population-based pregnancy cohort that recruited pregnant women in Norway between 1999 and 2008 at their routine ultrasound examination at gestational week 17–18.^20^ The initial participation rate was 41%. The cohort now includes 114 500 children. Mothers completed questionnaires at regular intervals during the pregnancy (gestational ages 17, 22, and 30 weeks) and after the child was born (6 months, 18 months, 3 years, and 5 years of age). MoBa data were linked to the Medical Birth Registry of Norway (MBRN) via the woman’s personal identification number. MBRN includes information on pregnancy, delivery, and neonatal health for all births in Norway.^21^ The MoBa was approved by the Regional Committee for Medical Research Ethics and the Norwegian Data Inspectorate.

**FIGURE 1:**
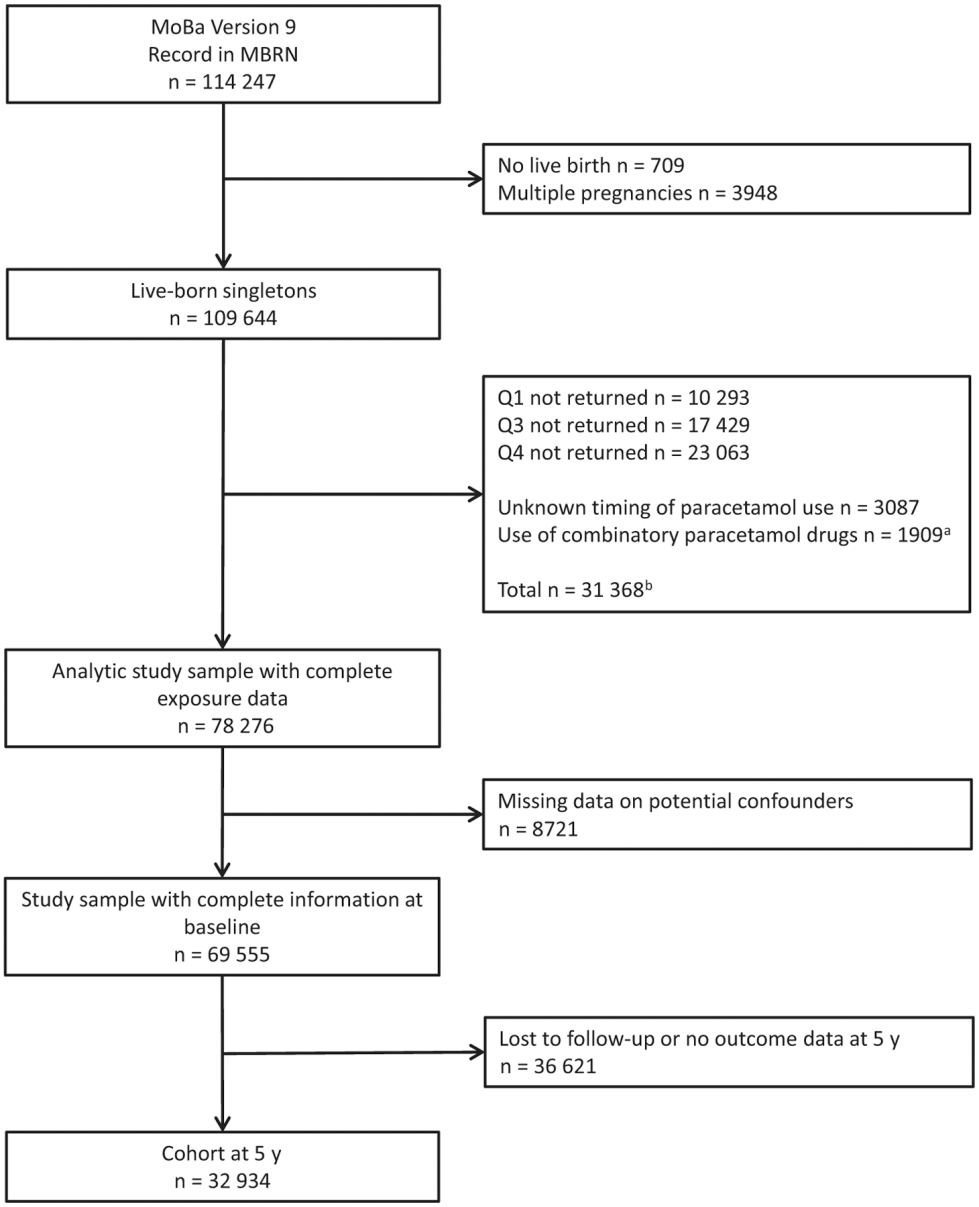
Participant flow chart. ^a^Use of drugs with ATC code N02BE51 or N02AA59. ^b^Conditions may overlap.Abbreviation: y, years

This study used data from the MoBa study (Data version 9, released 2015). We included women who had completed the questionnaires with information on medication exposure in pregnancy at GWs 17 and 30 (Q1, Q3) and 6 months postpartum (Q4). Women who used combination drugs including paracetamol were excluded in order to enable us to study the impact of paracetamol in itself. Figure 1 shows an overview of dropout and exclusion criteria. The study sample with complete information at baseline included 69 555 children, of which 32 934 (47.3%) had outcome data at 5 years. A comparison of the study sample with full cohort is given in Table S1, including the amount of missingness for each covariate. A comparison of exposure rates and characteristics of the mother-child pairs with the outcome measured and those lost to follow-up are given in Table S2.

### 2.2 | Paracetamol exposure

Information about medication use was obtained from two prenatal and one postnatal questionnaire. Women were presented with a list of indications where they could report the name of the medication taken in an open textbox along with timing of use (6 months prepregnancy, GW 0–4, 5–8, 9–12, 13+ (Q1), 13–16, 17–20, 21–24, 25–28 and 29+ (Q3), and week 30 until delivery (Q4)) and for how many days they had used it, according to a specific indication (eg “back pain,” “pelvic girdle pain,” and “headache”).

All medications were coded according to the Anatomical Therapeutic Chemical (ATC) Classification System.^22^ Paracetamol exposure was defined as the use of a medication with ATC code N02BE01. In Norway, paracetamol is available both over-the-counter and by prescription, and is the first-line analgesic in pregnancy. In the primary analysis, we explored the durational effects of prenatal paracetamol exposure. Duration of paracetamol use was defined according to the number of trimesters it was used: (a) paracetamol use in one trimester, (b) paracetamol use in two trimesters, (c) paracetamol use in three trimesters, and (d) no use during pregnancy (mutually exclusive groups). Within these categories, we explored the average number of days of paracetamol use. As a secondary analysis, we explored the effect of timing (first-trimester exposure (yes/no) and 2nd-3rd trimester exposure (yes/no)). Women who used paracetamol prior to pregnancy only constituted the negative control group. A table showing various patterns of paracetamol exposure can be found in the Table S3.

### 2.3 | Neurodevelopmental outcomes

Communication skills were assessed by the Ages and Stages Questionnaire (ASQ), which is considered to be an effective screening tool for detecting developmental delays. The communication domain consists of seven questions regarding the child’s language competence,^23^ and mothers answered “Yes,” “A few times,” or “Not yet” to statements according to whether the child could do the activity. Mean scores were calculated and standardised for all children with a response to at least six of the seven items on the scale. Communication problems were defined as children with T scores ≥65.^24^

Selected items from The Child Behaviour Checklist (CBCL) for preschool children (CBCL/1.5–5) was used to assess children’s behaviour.^25^ The CBCL/1.5/5 has several subscales (attention problems, aggressive behaviour, emotionally reactive, anxious/depressed, and somatic complaints) which are combined with 2 aggregated scales measuring externalising (the first 2 subscales) and internalising behaviour (the last 3 subscales). Mothers reported the extent to which they agreed with the behaviour statements using the response categories “Not true,” “Somewhat or sometimes true,” or “Very true or often true.” Mean scores were calculated and standardised for all children with complete outcome data. Children with T scores ≥63 were classified as having clinically significant externalising or internalising behaviour problems.^26^

Temperament was assessed by the short version of the Emotionality, Activity and Shyness Temperament Questionnaire (EAS), which measures the four temperament dimensions emotionality, activity, sociability, and shyness.^27,28^ Mothers reported how well the statements applied to their child’s behaviour using a five-response Likert scale ranging from “Not at all typical” to “Very typical.” As these are temperamental traits, akin to normal personality traits, there is no recommended cut-off. Higher T scores indicate children who are more emotional, more active, more sociable, or more shy.

All outcomes were parent-reported when the child was 5 years old. Additional information about items comprising the scales and Cronbach’s α can be found in the supplementary material and Table S4.

### 2.4 | Covariates

Potential confounders and risk factors for the outcomes were identified through a literature review and directed acyclic graphs (Figure S1).^29^ We included maternal age at delivery, marital status, education level, parity, pre-pregnancy body mass index (BMI), folic acid supplement, smoking habits, alcohol use, symptoms of anxiety and depression (measured by a short version of the Hopkins Symptoms Checklist (SCL-5)^30^), maternal health conditions during pregnancy, concomitant medication use, and child sex as covariates in the analysis. An overview of the sources of the covariates is provided in Table S5. Additional and more detailed information on the covariates can be found in the Supplementary Material.

### 2.5 | Statistical analysis

To account for measured differences between the women who used paracetamol during pregnancy and those who did not, we used propensity scores (PS) to calculate inverse probability of treatment weights (IPTW).^31^ All PS models were fit using logistic regression to estimate the probability of taking paracetamol in one trimester (model 1), two trimesters (model 2), and three trimesters (model 3) versus no use, respectively, conditional on measured confounders. We also fit PS models to estimate the probability of paracetamol use in the first trimester versus no use in the first trimester (model 4), and paracetamol use in the second/third trimester, but not in the first trimester versus no use during pregnancy (model 5), both conditional on measured confounders. Stabilised IPTW were calculated based on the estimated PS and the balance assessed by standardised differences (Table S6). A standardised difference <0.1 was considered acceptable.^31^ Two interaction terms were included in the third model (pain conditions by headache/migraine and depression scores by headache/migraine) to ensure sufficient balance between covariates.

To account for loss to follow-up at 5 years, we estimated stabilised inverse probability of censoring weights (IPCW), up-weighting the women who remained to represent similar women who dropped out from the baseline sample (n = 69 555).^32^ These weights included the same variables as the PS models, except that the interaction terms were removed from model 3. Characteristics of the weights are presented in Table S7. We fit outcome models with combined weights (IPTW × IPCW). Generalised linear models (with a negative binomial distribution and log link) and linear models were used to evaluate categorical outcomes (ASQ and CBCL) and continuous outcomes (EAS), respectively. Robust standard errors were used to calculate 95% confidence intervals (CIs).

We carried out multiple analyses to assess unmeasured confounding. First, we estimated the association between our negative control group and neurodevelopmental outcomes.^33,34^ Second, we investigated the treatment effect within different percentiles of the PS^35^ and asymmetrically trimmed the range of the PS^36^ for our main findings. Third, we used the bounding factor analysis to assess the impact of unmeasured confoudning.^37^

Sensitivity analyses investigating the association between prenatal paracetamol exposure and neurodevelopmental outcomes within different indications, analyses restricted to term pregnancies, a principal component analysis, and a probabilistic bias analysis can be found in the Supplementary Material. All methods are described in more detail in the Supplementary Material.

Stata MP version 14.1 was used for all statistical analyses.

## 3 | RESULTS

Among the 32 934 children who had outcome data at 5 years, 15 126 (45.9%) were born to mothers who had used paracetamol at least once during the pregnancy, and the most common indications for use were pain conditions, headache or migraine, and fever or infection. Overall, 8374 (25.4%), 4961 (15.1%), and 1791 (5.4%) women took paracetamol in one, two, or three trimesters, respectively. Within these categories, the average number of days reported was 3, 9, and 24, respectively. Characteristics of mother-child pairs are presented in Table 1. Women who used paracetamol during pregnancy were less likely to be first-time mothers, used co-medications more frequently, had more health problems, smoked more, and reported a low to moderate intake of alcohol more often than unexposed women.

### 3.1 | Neurodevelopmental outcomes

The prevalence of outcomes in the 5-year cohort was 7.5% for communication problems, 9.8% for externalising behavioural problems, and 10.3% for internalising behavioural problems. We found an increased risk of internalising (adjusted relative risk (RR) 1.36, 95% CI 1.02, 1.80) and externalising behaviour problems (RR 1.22, 95% CI 0.93, 1.60) in children whose mothers used paracetamol in three trimesters compared to unexposed children (Table 2). Children born to mothers who used paracetamol in two trimesters scored lower on shyness than unexposed children (adjusted β −0.62, 95% CI −1.05, −0.19; Table 3). We found no association between timing of paracetamol use during pregnancy and the outcomes examined (Tables 4 and 5). However, children exposed to paracetamol in 2nd/3rd trimester scored lower on shyness than unexposed children (adjusted β −0.32, 95% CI −0.66, 0.02).

### 3.2 | Assessment of unmeasured confounding

In the negative control analysis, 2843 women used paracetamol prior to pregnancy only, and 14 965 women were unexposed during pregnancy. Paracetamol use before pregnancy only was associated with communication problems (RR 1.19, 95% CI 1.02, 1.38) and lower activity levels in children (β −0.80, 95% CI −1.23, −0.36) in adjusted models (Tables S8 and S9).

We observed a non-uniform treatment effect across different strata of the PS for the effect of paracetamol exposure in three trimesters on internalising behaviour and the effect of paracetamol exposure in two trimesters on shyness (Tables S10 and S11). Asymmetric trimming resulted in slightly reduced effect estimates for internalising behaviour, but not for shyness (Tables S12 and S13). A closer investigation of women exposed to paracetamol in three trimesters who also were in the low tail of the PS (n = 11) revealed that these women used paracetamol with high frequency and reported more offspring internalising problems, but did not report using paracetamol for any of the most common indications.

The bounding factor analysis showed that confounding of strength equal to an RR of 2.06 (on both sides) could completely explain away an observed RR of 1.36 between paracetamol use in three trimesters and internalising behaviour problems, but a weaker confounder could not.

Additional results are available in the Supplementary Material.

## 4 | COMMENT

### 4.1 | Principal findings

In our primary analyses, according to duration of paracetamol exposure we found a moderate increased risk of internalising behaviour and a borderline increased risk of externalising behaviour in children exposed to paracetamol in three trimesters compared with unexposed children. Children exposed to paracetamol in two trimesters scored lower on shyness than unexposed children, but the difference in mean T scores was small (50.1 vs 49.8). In secondary analyses by timing of exposure, we found a small borderline association between exposure to paracetamol in the 2nd/3rd trimester and lower shyness, which is in line with findings from the duration analysis. Even though disentangling the effect of duration from timing is challenging, the effect estimates for shyness were in the same direction, albeit the latter estimate was of smaller magnitude. Sensitivity analyses indicated that unmeasured confounding plays an important role and we cannot rule out chance or unmeasured confounding as possible explanations for our findings.

### 4.2 | Strengths of the study

By using data from the MoBa study, we have the unique opportunity to study the potential long-term effects of medications in pregnancy due to its large sample size, prospective design, and long follow-up. The MoBa provides detailed information on a range of variables, including maternal sociodemographic and lifestyle factors, medication use, and indications of use. An important strength of our study was that we were able to adjust for the indication of use, which is important given that some of the indications for which paracetamol is used may have effects on foetal health.^38^ Furthermore, we used advanced statistical methods to control for important confounders and performed a robust set of additional analyses to investigate the role of unmeasured confounding, as well as other sources of bias.

### 4.3 | Limitations of the study

The MoBa has a low participation rate with a possibility of self-selection of the healthiest women. Prior studies have shown that prevalence estimates may not be generalisable; however, the measures of tested associations were valid in MoBa.^39^ Although we used IPCWs to account for loss to follow-up at 5 years, we cannot rule out that selection bias may have affected our results. Both exposure and outcomes were parent-reported and subject to misclassification. Probabilistic bias analysis revealed that non-differential exposure misclassification may have resulted in underestimating the true exposure effects. On the other hand, dependent misclassification is possible.^40^ Importantly, it is likely that biases from misclassification and confounding act jointly, but in opposite directions, and our results should be interpreted with this in mind. No information on formulation or dose was available; however, we examined days of use in order to get a better understanding of exposure duration.

### 4.4 | Interpretation

This study is a follow-up of the MoBa and adds to the current literature on long-term neurodevelopment of children prenatally exposed to paracetamol by more closely exploring the role of unmeasured confounding. It is reassuring that the use of paracetamol in one trimester was not associated with communication, behavioural, or temperamental problems in children 5 years of age and also that timing of paracetamol use during pregnancy does not seem to increase the risk of the outcomes examined. Furthermore, paracetamol exposure during pregnancy did not seem to have a negative impact on communication skills among preschool-aged children.

Across the lifespan, shyness is associated with a variety of social and emotional problems, particularly along the internalising dimension.^41^ Our association between prenatal paracetamol exposure and less shyness in children was not due to low levels of positive emotionality (ie low extraversion and low activity), but was specific to shyness. This may indicate a more undifferentiated expression of feelings among the children.^41^ In novel situations, a moderate fear of strangers is normative for preschool-aged children and the effect could represent dysregulated behaviour, but the clinical meaning of this finding is uncertain.

Earlier publications from the MoBa found an association between prenatal paracetamol exposure for 28 days or more, and communication problems, externalising and internalising behaviour problems, and higher activity levels in 3-year-old children.^1^ Communication problems were also present at 18 months.^8^ After 5 years of follow-up, only internalising behaviour problems remained. We could not replicate the association between long-term prenatal exposure to paracetamol and communication or activity problems observed in younger children. An explanation for the different findings may be that problems detected in early childhood have resolved by 5 years of age because symptoms of emotional and behavioural problems may change or evolve as a child grows older.^42^ We must also keep in mind that some problems are detected more easily when the child is older; therefore, it is important to re-assess neurodevelopmental outcomes in children after a longer follow-up period.^15^ When comparing our exposure definition with prior studies,^1,8^ 56.4% of the women reporting use of paracetamol for more than 28 days, were classified as exposed in three trimesters in our study.

### 4.5 | Bias from unmeasured confounding

If there is a causal effect of paracetamol exposure during pregnancy on child neurodevelopment, we would expect a null finding in the negative control analysis as paracetamol used prior to pregnancy cannot directly impact neurodevelopment. However, we found positive associations between our negative control group^34^ and some child outcomes, though different outcomes than those identified in the main analyses. This indicates that there is unmeasured confounding and our observed associations may be confounded to some extent by unobserved maternal factors, such as personality traits^43^ or genetics. There could be unobserved factors related to analgesic use and adherence during pregnancy that cause the observed observations. Using a similar methodological approach, Harris et al^44^ recently found an unexpected association between maternal triptan use during pregnancy and offspring sociability at 5 years. Moreover, the non-uniform treatment effect across the PS supports the presence of unmeasured confounding.^35,36^ Asymmetric trimming could not fully wash away the observed associations, but the effect estimate of paracetamol use in three trimesters on internalising behaviour was reduced and further attenuated when we excluded women in the low tail of the PS (n = 11). The bounding factor analysis showed that only a strong confounder can fully explain away the observed exposure-outcome association. Given the magnitude of the association between high contentiousness and use of paracetamol during pregnancy (odds ratio 0.74 (95% CI 0.55, 0.99),^43^ maternal personality traits may not fully explain our finding. However, these analyses suggest that unmeasured confounding plays an important role and may, at least in part, possibly explain our results.

In this study, we examined three important domains of neurodevelopment, namely communication skills, behaviour, and temperament by using screening instruments widely recognised within child psychiatry and psychology.^24,26,27^ These tools show high internal consistency and are strongly predictive of later child diagnosis.^23,26,28^ As MoBa is an ongoing study, future studies should describe trajectories of early childhood problems and their association with later diagnosis. Moreover, there is a need for international authoritative guidance on how to measure neurodevelopmental outcomes in medication safety in pregnancy studies.^45^

## 5 | CONCLUSIONS

Overall, paracetamol use as short term or at different timing in pregnancy does not seem to have a negative impact on child communication, behaviour, or temperament in preschool-aged children. Children exposed to paracetamol in two trimesters scored lower on shyness, and children exposed to paracetamol in three trimesters had a moderate increased risk of internalising behaviour problems compared with unexposed children. However, some evidence suggests that unmeasured confounding could possibly explain these findings. Pregnant women should be empowered to make appropriate decisions about their use of over-the-counter analgesics such as paracetamol during pregnancy to avoid both overuse and under use of over-the-counter analgesics and avoid unfounded concerns about the risks of paracetamol to the unborn child.

## Supplementary Material

Supplementary material

## Figures and Tables

**TABLE 1:** Maternal and child characteristics of the 5-year cohort (n = 32 934) according to paracetamol exposure during pregnancy

	No use of paracetamol during pregnancy (n = 17 808)	Paracetamol use in one trimester (n = 8374)	Paracetamol use in two trimesters (n = 4961)	Paracetamol use in three trimesters (n = 1791)
Maternal characteristics				
Mean age at time of delivery, years (SD)	30.8 (4.4)	30.4 (4.3)	30.5 (4.3)	30.8 (4.3)
Married/cohabiting, n (%)	17 215 (96.7)	8104 (96.8)	4807 (96.9)	1748 (97.6)
Primiparous, n (%)	9113 (51.2)	4072 (48.6)	2130 (42.9)	638 (35.6)
University/college education, n (%)	13 738 (77.2)	6385 (76.3)	3772 (76.0)	1348 (75.3)
Mean pre-pregnancy BMI, kg/m^2^ (SD)	23.5 (3.9)	23.9 (4.1)	24.4 (4.3)	24.9 (4.7)
Folic acid supplement, n (%)	15 190 (85.3)	7216 (86.2)	4346 (87.6)	1588 (88.7)
Symptoms of anxiety/depression^a^, z score (SD)	−0.09 (0.8)	−0.01 (0.9)	0.04 (0.9)	0.18 (1.0)
Smoking during pregnancy, n (%)				
No	14 726 (82.7)	6632 (79.2)	3931 (79.2)	1414 (79.0)
Yes	680 (3.8)	412 (4.9)	266 (5.4)	85 (4.8)
Stopped	2402 (13.5)	1330 (15.9)	764 (15.4)	292 (16.2)
Alcohol intake during pregnancy, n (%)				
No or minimal	15 789 (88.7)	7305 (87.2)	4357 (87.8)	1526 (85.2)
Low to moderate	1851 (10.4)	972 (11.6)	568 (11.5)	239 (13.3)
Frequent	168 (0.9)	97 (1.2)	36 (0.7)	26 (1.5)
Health conditions, n (%)				
Headache or migraine	3257 (18.3)	3202 (38.2)	3137 (68.2)	1399 (78.1)
Pain^b^	11 093 (62.3)	5908 (70.6)	3719 (75.0)	1451 (81.0)
Fever or infections	4244 (23.8)	3535 (42.2)	2182 (44.0)	806 (45.0)
Co-medications, n (%)				
NSAIDs (M01A, N02BA)	629 (3.5)	680 (8.1)	558 (11.3)	322 (18.0)
Opioids (N02A)	14 (0.1)	20 (0.2)	15 (0.3)	13 (0.7)
Psychotropic drugs^c^	371 (2.1)	220 (2.6)	152 (3.1)	78 (4.4)
Triptans (N02CC)	66 (0.4)	64 (0.8)	(100 (2.0)	61 (3.4)
Child characteristics				
Boy, n (%)	9198 (51.7)	4208 (50.3)	2523 (50.9)	861 (48.1)
Preterm^d^ (<37 weeks), n (%)	753 (4.3)	377 (4.5)	210 (4.3)	69 (3.9)
Low birthweight^d^ (<2500 g), n (%)	402 (2.3)	225 (2.7)	139 (2.8)	32 (1.8)
Malformations^d^, n (%)	881 (5.0)	385 (4.6)	245 (4.9)	86 (4.8)

a Measured by a short version of the Hopkins Symptoms Checklist (SCL-5) in Q1 and Q3.

b Includes back pain, neck and shoulder pain, pelvic girdle pain, and other pains in muscle/joints.

c Psychotropic drugs were further divided into the following groups in the statistical analyses: antidepressants (N06A), antipsychotics (N05A), antiepileptics (N03A), stimulants (N06BA), benzodiazepines (N05BA, N05CD), and benzodiazepine-like drugs (N05CF).

d Not included in IPT weighting based on DAG.

**TABLE 2:** Associations between duration of paracetamol exposure during pregnancy and communication and behavioural problems in preschool-aged children

Communication and behavioural problems^a^	Total n	Percentage with outcome	Unadjusted RR (95% CI)	Adjusted RR (95% CI)
Communication problems				
Never user	17 317	7.4	1.00 (Reference)	1.00 (Reference)
Paracetamol use in one trimester	8180	7.4	1.00 (0.91, 1.09)	0.98 (0.88, 1.09)
Paracetamol use in two trimesters	4835	7.4	1.00 (0.89, 1.11)	0.85 (0.73, 1.00)
Paracetamol use in three trimesters	1757	8.5	1.15 (0.98, 1.35)	1.18 (0.86, 1.60)
Externalising problems				
Never user	17 283	9.4	1.00 (Reference)	1.00 (Reference)
Paracetamol use in one trimester	8136	10.1	1.08 (1.00, 1.17)	1.03 (0.95, 1.14)
Paracetamol use in two trimesters	4823	10.0	1.06 (0.97, 1.17)	1.00 (0.87, 1.14)
Paracetamol use in three trimesters	1742	12.5	1.33 (1.17, 1.52)	1.22 (0.93, 1.60)
Internalising problems				
Never user	17 446	9.8	1.00 (Reference)	1.00 (Reference)
Paracetamol use in one trimester	8213	10.7	1.09 (1.01, 1.18)	1.03 (0.95, 1.13)
Paracetamol use in two trimesters	4857	10.3	1.04 (0.95, 1.15)	0.92 (0.81, 1.05)
Paracetamol use in three trimesters	1754	12.7	1.29 (1.13, 1.47)	1.36 (1.02, 1.80)

*Note:* Adjusted estimates are weighted with combined weights (IPTW × IPCW).

Abbreviations: RR, relative risk; CI, confidence interval.

a Communication skills were assessed by the ASQ and behaviour problems by the CBCL.

**TABLE 3:** Associations between duration of paracetamol exposure during pregnancy and temperamental traits in preschool-aged children

Temperament^a^	Total n	Mean T score (SD)	Unadjusted β (95% CI)	Adjusted β (95% CI)
Emotionality				
Never user	17 416	49.7 (10.0)	0.00 (Reference)	0.00 (Reference)
Paracetamol use in one trimester	8228	50.1 (9.9)	0.31 (0.05, 0.57)	0.24 (−0.06, 0.53)
Paracetamol use in two trimesters	4858	50.2 (9.9)	0.46 (0.14, 0.77)	−0.01 (−0.44, 0.41)
Paracetamol use in three trimesters	1756	50.6 (10.1)	0.81 (0.31, 1.30)	0.13 (−1.08, 1.33)
Activity				
Never user	17 612	49.9 (10.0)	0.00 (Reference)	0.00 (Reference)
Paracetamol use in one trimester	8303	49.9 (10.0)	0.03 (−0.23, 0.29)	−0.08 (−0.38, 0.21)
Paracetamol use in two trimesters	4901	49.9 (9.9)	−0.02 (−0.33, 0.29)	−0.04 (−0.48, 0.39)
Paracetamol use in three trimesters	1771	50.1 (10.2)	0.25 (−0.25, 0.75)	0.51 (−0.57, 1.60)
Sociability				
Never user	17 604	50.0 (9.9)	0.00 (Reference)	0.00 (Reference)
Paracetamol use in one trimester	8298	50.0 (9.9)	0.06 (−0.20, 0.32)	0.02 (−0.27, 0.32)
Paracetamol use in two trimesters	4908	50.2 (10.0)	0.23 (−0.09, 0.54)	0.30 (−0.12, 0.73)
Paracetamol use in three trimesters	1777	50.1 (9.8)	0.03 (−0.45, 0.51)	−0.07 (−1.02, 0.88)
Shyness				
Never user	17 512	50.1 (10.0)	0.00 (Reference)	0.00 (Reference)
Paracetamol use in one trimester	8252	50.0 (9.9)	−0.10 (−0.36, 0.16)	−0.17 (−0.46, 0.13)
Paracetamol use in two trimesters	4874	49.8 (9.8)	−0.30 (−0.61, 0.01)	−0.62 (−1.05, −0.19)
Paracetamol use in three trimesters	1760	50.0 (10.0)	−0.07 (−0.56, 0.42)	−0.24 (−1.27, 0.80)

*Note:* Adjusted estimates are weighted with combined weights (IPTW × IPCW).

Abbreviations: SD, standard deviation; CI, confidence interval.

a Temperamental traits were assessed by the EAS.

**TABLE 4:** Associations between timing of paracetamol exposure and communication and behavioural problems in preschool-aged children

Communication and behavioural problems^a^		Total n	Percentage with outcome	Unadjusted RR (95% CI)	Adjusted RR (95% CI)
Communication problems					
Paracetamol use in 1st trimester	No	23 706	7.4	1.00 (Reference)	1.00 (Reference)
	Yes	8383	7.6	1.03 (0.94, 1.12)	0.98 (0.88, 1.08)
Paracetamol use in 2nd/3rd trimester^b^	No	17 317	7.4	1.00 (Reference)	1.00 (Reference)
	Yes	6389	7.4	1.00 (0.90, 1.10)	0.97 (0.86, 1.10)
Externalising problems					
Paracetamol use in 1st trimester	No	23 632	9.7	1.00 (Reference)	1.00 (Reference)
	Yes	8352	10.3	1.06 (0.99, 1.14)	0.99 (0.91, 1.08)
Paracetamol use in 2nd/3rd trimester^b^	No	17 283	9.4	1.00 (Reference)	1.00 (Reference)
	Yes	6349	10.5	1.12 (1.02, 1.21)	1.09 (0.98, 1.20)
Internalising problems					
Paracetamol use in 1st trimester	No	23 859	10.0	1.00 (Reference)	1.00 (Reference)
	Yes	8411	11.1	1.10 (1.02, 1.19)	0.99 (0.91, 1.07)
Paracetamol use in 2nd/3rdtrimester^b^	No	17 446	9.8	1.00 (Reference)	1.00 (Reference)
	Yes	6413	10.5	1.06 (0.98, 1.16)	1.05 (0.95, 1.16)

*Note:* Adjusted estimates are weighted with combined weights (IPTW × IPCW).

Abbreviations: RR, relative risk; CI, confidence interval.

a Communication skills were assessed by the ASQ and behaviour problems by the CBCL.

b Paracetamol use in 2nd and/or 3rd trimester, but not in 1st trimester.

**TABLE 5:** Associations between timing of paracetamol exposure and temperamental traits in preschool-aged children

Temperament^a^		Total n	Mean T score (SD)	Unadjusted β (95% CI)	Adjusted β (95% CI)
Emotionality					
Paracetamol use in 1st trimester	No	23 828	49.8 (9.9)	0.00 (Reference)	0.00 (Reference)
	Yes	8430	50.3 (10.0)	0.42 (0.18, 0.67)	0.16 (−0.12, 0.44)
Paracetamol use in 2nd/3rd trimester^b^	No	17 416	49.8 (10.0)	0.00 (Reference)	0.00 (Reference)
	Yes	6412	50.1 (9.9)	0.30 (0.02, 0.59)	0.21 (−0.12, 0.55)
Activity					
Paracetamol use in 1st trimester	No	24 085	49.9 (10.0)	0.00 (Reference)	0.00 (Reference)
	Yes	8502	49.9 (10.0)	−0.05 (−0.29, 0.20)	−0.03 (−0.32, 0.26)
Paracetamol use in 2nd/3rd trimester^b^	No	17 612	49.9 (10.0)	0.00 (Reference)	0.00 (Reference)
	Yes	6473	50.0 (10.0)	0.11 (−0.16, 0.40)	−0.02 (−0.36, 0.32)
Sociability					
Paracetamol use in 1st trimester	No	24 066	50.0 (9.9)	0.00 (Reference)	0.00 (Reference)
	Yes	8521	50.2 (9.9)	0.17 (−0.07, 0.41)	0.21 (−0.07, 0.49)
Paracetamol use in 2nd/3rd trimester^b^	No	17 604	49.9 (9.9)	0.00 (Reference)	0.00 (Reference)
	Yes	6462	50.0 (9.9)	0.02 (−0.25, 0.31)	0.00 (−0.33, 0.35)
Shyness					
Paracetamol use in 1st trimester	No	23 952	50.0 (9.9)	0.00 (Reference)	0.00 (Reference)
	Yes	8445	50.0 (10.0)	−0.05 (−0.30, 0.20)	−0.17 (−0.45, 0.11)
Paracetamol use in 2nd/3rd trimester^b^	No	17 512	50.0 (10.0)	0.00 (Reference)	0.00 (Reference)
	Yes	6441	49.9 (9.8)	−0.23 (−0.51, 0.05)	−0.32 (−0.66, 0.02)

*Note:* Adjusted estimates are weighted with combined weights (IPTW × IPCW).

Abbreviations: SD, standard deviation; CI, confidence interval.

a Temperamental traits were assessed by the EAS.

b Paracetamol use in 2nd and/or 3rd trimester, but not in 1st trimester.

## SYNOPSIS

### Study question

We investigated the association between prenatal paracetamol exposure and parent-reported communication skills, behavioural, and temperamental problems in preschool-aged children and explored the role of unmeasured confounding.

### What’s already known

Recent studies have suggested an association between prenatal paracetamol exposure and adverse neurodevelopmental outcomes in children. Given the widespread use of paracetamol during pregnancy, establishing its long-term neurodevelopmental safety is of great public health interest.

### What this study adds

We found no substantial associations between timing of prenatal paracetamol exposure on the outcomes examined. Paracetamol use in multiple trimesters was associated with lower shyness and greater internalising behaviour in preschool-aged children. However, we cannot rule out chance or confounding by unmeasured factors as possible explanations for our findings.
