# Supplementary material for: Prenatal paracetamol exposure and neurodevelopmental outcomes in preschool-aged children
Source: Paediatr Perinat Epidemiol. Author manuscript; Available in PMC 2021 Jul 16. (PMC8285062; doi:10.1111/ppe.12568)
Supplement: Supplementary material [file NIHMS1590632-supplement-Supplementary_material.docx]

**Supplemental material**

Complete questionnaires and instrument documentation are available at: <https://www.fhi.no/en/studies/moba/for-forskere-artikler/questionnaires-from-moba/>

**Additional information on “Methods”**

Neurodevelopmental outcomes

Items comprising each scale and reliability coefficients are presented in eTable 4.

*ASQ:* The ASQ is considered to be an effective screening tool for detecting developmental delays, however, brief scales are prone to unreliability, leading to attenuation of true effects. This could lead to negative findings of true small effects in the population.

The scale consists of the following 5 domains; communication, gross motor, fine motor, problem solving and personal-social.^1^ In MoBa Q5-years only the communication domain is included. The communication domain consists of seven questions regarding the child’s language competence. There are six original items and one item that was adapted from the 4-year questionnaire to increase reliability and sensitivity to very low levels of communication skills at 5 years.

*CBCL:* The CBCL version for preschool children (CBCL/1.5-5) is a widely used and validated measure of children’s behaviour, and covers a range of emotional, social, and behavioural problems.^2^ The CBCL version for older children has been validated in a Norwegian sample,^3^ whereas the CBCL1.5/5 has been validated in Dutch and Danish samples.^4,5^ The original instrument consists of 100 items describing a behaviour exhibited by the child during the last two months. Due to space restrictions, the full version was not included in the MoBa questionnaires. Therefore, specifically selected items that were intended to represent all CBCL subscales, and to be clinically and theoretically relevant indications of behaviour problems were included in the MoBa.

*EAS:* EAS measures the four temperament dimensions: emotionality (the tendency to become emotionally aroused easily and intensely), activity (preferred activity level), sociability (the tendency to prefer the presence of others to being alone), and shyness (fear of strangers, social inhibition).

Covariates

An overview of the sources of the covariates is presented in eTable 5.

*Education level:* Education level was coded as the highest level of completed or ongoing education.

*Folic acid supplement:* Folic acid supplement was coded as folic acid intake before and during pregnancy (4 weeks prior to pregnancy and/or until week 12 of pregnancy).

*Symptoms of anxiety/depression:* Symptoms of anxiety/depression were measured by a short version of the Hopkins Symptoms Checklist (SCL-5) twice during pregnancy.^6^ The SCL is a psychometric scale designed to screen for symptoms of depression in population surveys.^7^ Mean scores were calculated at each time point and standardised.

*Alcohol use during pregnancy:* Alcohol intake was classified as “No or minimal” (less than once per month), “Moderate” (once per month to once per week), and “Frequent” (more than once per week).

*Maternal health conditions:* We included the following maternal health conditions: fever, headache/migraine, infections (respiratory, genital, and urinary), and pain conditions (back pain, pelvic girdle pain, neck and shoulder pain, and other pain in muscle/joints) as covariates in our analyses.

*Co-medications:* Assessed co-medications included non-steroidal anti-inflammatory drugs (NSAIDs) (ATC code M01A (e.g. diclofenac, ibuprofen, and naproxen) and N02BA (aspirin = acetylsalicylic acid)), opioids (N02A), triptans (N02CC), antiepileptics (N03A), anti-psychotics (N05A), antidepressants (N06A), benzodiazepines (N05CD and N05BA), benzodiazepine-like drugs (N05CF), and stimulants (N06BA). The latter six ATC codes were merged into one category of “psychotropic drugs” for ease of presentation in tables.

Data analysis

*Negative control:* We used women exposed to paracetamol in the 6-month period prior to pregnancy but not during pregnancy as a negative control group. These women were compared to women who were not exposed to paracetamol during pregnancy. Using logistic regression, we estimated the probability of being exposed to paracetamol prior to pregnancy compared to not using paracetamol during pregnancy, conditional on maternal age, marital status, parity, education level, pre-pregnancy BMI, smoking and alcohol use before pregnancy, and use of co-medications in the 6-month period before pregnancy (ATC codes: M01A, N02BA, N02A, N02CC, N03A, N05A, N06A, N05CD, N05BA, N05CF, and N06BA). The PS was then used to calculate stabilised IPTW, and the weights were sufficiently balanced. Generalised and linear models with robust standard errors were used to assess categorical outcomes (ASQ and CBCL) and continuous outcomes (EAS).

*Investigating women in the tails of the PS and asymmetric trimming:* We investigated the effect of paracetamol exposure in three trimesters on internalising behaviour according to percentiles of the PS. Next, we asymmetrically trimmed the range of PS of never user vs, user of paracetamol in three trimesters corresponding to the 1^st^ and 99^th^ percentiles, the 2.5^th^ and 97.5^th^ percentiles, the 5^th^ and 95^th^ percentiles, and the 10^th^ and 90^th^ percentiles of the PS distribution in the exposed and unexposed women, respectively, to reduce bias by unmeasured confounding.^8^ These analyses were repeated for women using paracetamol in two trimesters and shyness.

*Stratified analyses:* We investigated the association between prenatal paracetamol exposure and neurodevelopmental outcomes within different indications (headache/migraine, fever, and pain) to assess the role of confounding by indication.

*Restricting to term pregnancies:* We repeated our main analysis restricting it to women with term pregnancies because preterm birth is a risk factor for the outcome.

*Probabilistic bias analysis of exposure misclassification:* We investigated the effect of exposure misclassification on the association between paracetamol use in three trimesters and internalising problems by specifying a trapezoidal distribution of values for the sensitivity and specificity of paracetamol use. The sensitivity was reported to be 0.57 (95% CI 0.51, 0.64) by van Gelder et al^9^; therefore, we set our modes to the following values: 0.50 – 0.60 – 0.75 – 1.00. Specificity was reported to be high in the MoBa^10^; therefore, we set our modes of specificity to the following values: 0.90 – 0.94 – 0.98 – 1.00. We assumed that the exposure misclassification was non-differential and ran 10 000 simulations.

*Principal component analysis:* To assess general risk factors for a wide range of neurodevelopmental outcomes, we reduced the correlated scales (i.e., ASQ communication, CBCL externalising and internalising subscales, and EAS temperament) to a set of uncorrelated factor scores by principal component analysis with varimax rotation.^11^ Conceptually, these factors index overarching traits responsible for psychiatric comorbidity and association between temperament and psychopathology (e.g., negative and positive emotionality).^12^ We estimated the association between general traits for neurodevelopment and exposure and adjusted for the general trait factor scores to assess the extent to which the exposure-outcome association was specific to a neurodevelopmental outcome.

**Additional information on “Results”**

In analyses stratified by indication, effect estimates for paracetamol were generally similar to those of the main analysis, and no specific trends were observed (results not shown).

Repeating our main analysis by restricting the data to term pregnancies did not substantially affect our point estimates (results not shown).

The probabilistic bias analysis for misclassification of paracetamol exposure in three trimesters on internalising behaviour yielded a corrected RR of 1.57 (95% CI [1.25, 4.18]) compared to the conventional RR of 1.29 (95% CI [1.13, 1.47]). This indicates that we may have underestimated the true effect.

The principal component analysis revealed two broad factors reflecting negative and positive emotionality (eTable 14). Paracetamol exposure was not associated with these general factors after adjustment. The exposure-outcome association for shyness was not attenuated by adjusting for the general trait factors, but the exposure-outcome association for internalising behaviour no longer remained significant (results not shown).

**Supplementary figures**

[**eFigure 1.** Simplified directed acyclic graph (DAG) for the association of prenatal paracetamol exposure with neurodevelopment in the child](#_Toc8717396)


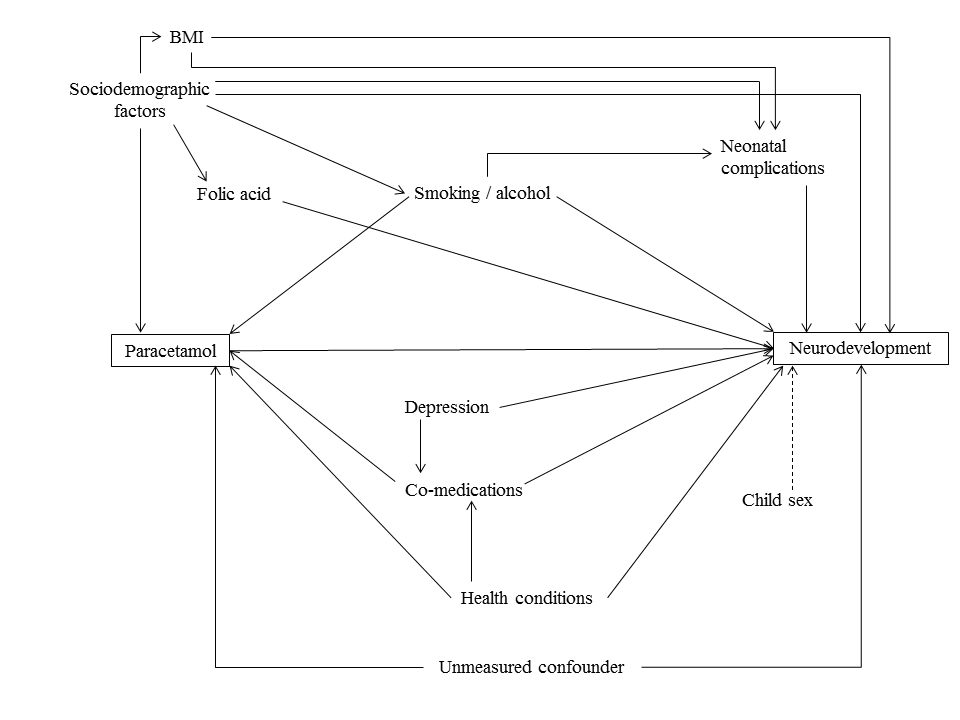
Sociodemographic factors include maternal age, parity, education, and marital status.
The unmeasured confounder may be unobserved maternal factors or genetics.
Health conditions include headache or migraine, fever, pain, and infections.
Neonatal complications include preterm birth, malformations, and low birthweight.
Solid lines are biasing paths, while dashed lines are risk factors for the outcome.

**eFigure 1.** Simplified directed acyclic graph (DAG) for the association of prenatal paracetamol exposure with neurodevelopment in the child

**Supplementary tables**

[**eTable 1.** Characteristics of the study sample, the analytic study sample, and the full MoBa cohort](#_Toc8717397)

[**eTable 2.** Characteristics of mother-child pairs for which the outcome was or was not measured at 5 years or were lost to follow-up](#_Toc8717398)

[**eTable 3.** Various patterns of paracetamol exposure during pregnancy in the 5-year cohort (n=32 934)](#_Toc8717399)

[**eTable 4.** Items composing the ASQ, CBCL, and EAS in MoBa Q-5year and sub-scale reliability information (Cronbach’s α)](#_Toc8717400)

[**eTable 5.** Overview of the sources of the relevant variables in the MoBa and MBRN.](#_Toc8717401)

[**eTable 6.** Balance of covariates between the exposed/unexposed groups](#_Toc8717402)

[**eTable 7.** Characteristics of generated stabilised weights](#_Toc8717403)

[**eTable 8.** Associations between exposure to paracetamol prior to pregnancy only and communication and behaviour problems in preschool-aged children](#_Toc8717404)

[**eTable 9.** Associations between exposure to paracetamol prior to pregnancy only and temperamental traits in preschool-aged children](#_Toc8717405)

[**eTable 10.** Proportion of internalising behavioural problems among children born to mothers who used paracetamol in three trimesters and children born to mothers who did not use paracetamol according to percentiles of the propensity score](#_Toc8717406)

[**eTable 11.** Shyness temperament among children born to mothers who used paracetamol in two trimesters and children born to mothers who did not use paracetamol according to percentiles of the propensity score](#_Toc8717407)

[**eTable 12.** Asymmetric trimming of the propensity score in analyses of the association between paracetamol exposure in three trimesters and internalising behaviour problems](#_Toc8717408)

[**eTable 13.** Asymmetric trimming of the propensity score in analyses of the association between paracetamol exposure in two trimesters and shyness traits](#_Toc8717409)

[**eTable 14.** Principal component analysis (PCA)](#_Toc8717410)

**eTable 1.** Characteristics of the study sample, the analytic study sample, and the full MoBa cohort

|  | Study sample  n=69 555 | Analytic study sample  n=78 276 | Full MoBa cohort  n=114 247 |
| --- | --- | --- | --- |
| Maternal characteristics |  |  |  |
| Mean age at time of delivery, years (SD) | 30.2 (4.5) | 30.2 (4.5) | 30.1 (4.7) |
| Married/cohabiting, % | 96.6 | 96.5 | 95.4 |
| Primiparous, % | 46.4 | 46.0 | 44.1 |
| University/college education, % | 70.1 | 68.5 | 60.1 |
| Missing | - | 0.4 | 9.8 |
| Mean pre-pregnancy BMI, kg/m^2^ (SD) | 24.0 (4.2) | 24.0 (4.2) | 24.1 (4.3) |
| Missing, % | - | 2.4 | 11.8 |
| Folic acid supplement, % | 78.4 | 77.1 | 68.5 |
| Symptoms of depression/anxiety,  z-score (SD) | 0.000 (0.9) | -0.003 (0.9) | -0.013 (0.9) |
| Missing, % | - | 2.8 | 19.8 |
| Smoking during pregnancy, % |  |  |  |
| No | 76.3 | 72.3 | 52.8 |
| Yes | 6.7 | 6.7 | 7.3 |
| Stopped | 17.0 | 16.7 | 15.4 |
| Missing | - | 4.3 | 24.5 |
| Alcohol intake during pregnancy, % |  |  |  |
| No or minimal | 87.3 | 85.1 | 71.2 |
| Low to moderate | 11.7 | 11.3 | 9.2 |
| Frequent | 1.0 | 1.0 | 0.7 |
| Missing | - | 2.6 | 18.9 |
| Health conditions, % |  |  |  |
| Headache or migraine | 33.5 | 33.3 | 31.0 |
| Pain | 68.1 | 67.7 | 59.6 |
| Fever or infections | 33.1 | 33.1 | 29.4 |
| Co-medications, % |  |  |  |
| NSAIDs (M01A, N02BA) | 6.8 | 6.7 | 6.2 |
| Opioids (N02A) | 0.2 | 0.2 | 1.9 |
| Psychotropic drugs^a^ | 2.6 | 2.7 | 2.8 |
| Triptans (N02CC) | 0.8 | 0.8 | 0.8 |
| Child characteristics |  |  |  |
| Boy, % | 51.2 | 51.2 | 51.2 |
| Missing | - | - | 0.2 |
| Preterm^b^ (< 37 weeks), % | 4.2 | 4.3 | 6.8 |
| Missing | 0.4 | 0.4 | 0.4 |
| Low birthweight^b^ (<2500 g), % | 2.4 | 2.4 | 4.6 |
| Missing | 0.1 | 0.1 | 0.2 |
| Congenital malformations^b^, % | 4.6 | 4.6 | 5.0 |
| ^a^ Psychotropic drugs include: antidepressants (N06A), antipsychotics (N05A), antiepileptics (N03A), stimulants (N06BA), benzodiazepines (N05BA, N05CD), and benzodiazepine-like drugs (N05CF).  ^b^ Variables are not included in the analysis. | | | |

**eTable 2.** Characteristics of mother-child pairs for which the outcome was or was not measured at 5 years or were lost to follow-up

|  | **Outcome measured at 5 years**  **n=32 934** | **No outcome measured at 5 years or lost to follow-up**  **n=36 621** |
| --- | --- | --- |
| Maternal characteristics |  |  |
| Mean age at time of delivery, years (SD) | 30.6 (4.3) | 29.9 (4.5) |
| Married/cohabiting, n (%) | 31 874 (96.8) | 35 318 (96.4) |
| Primiparous, n (%) | 15 953 (48.4) | 16 308 (44.5) |
| University/college education, n (%) | 25 243 (76.7) | 23 507 (64.2) |
| Mean pre-pregnancy BMI, kg/m^2^ (SD) | 23.8 (4.1) | 24.1 (4.3) |
| Folic acid supplement, n (%) | 28 340 (86.1) | 26 154 (71.4) |
| Symptoms of depression/anxiety, z-score (SD) | -0.04 (0.9) | 0.03 (0.9) |
| Smoking during pregnancy, n (%) |  |  |
| No | 26 703 (81.1) | 26 365 (72.0) |
| Yes | 1443 (4.4) | 3215 (8.8) |
| Stopped | 4788 (14.5) | 7041 (19.2) |
| Alcohol intake during pregnancy, n (%) |  |  |
| No or minimal | 28 977 (88.0) | 31 793 (86.8) |
| Low to moderate | 3630 (11.0) | 4484 (12.2) |
| Frequent | 327 (1.0) | 344 (1.0) |
| Health conditions, n (%) |  |  |
| Headache or migraine | 10 995 (33.4) | 12 325 (33.7) |
| Pain | 22 171 (67.3) | 25 187 (68.8) |
| Fever or infections | 10 767 (32.7) | 12 269 (33.5) |
| Co-medications, n (%) |  |  |
| NSAIDs (M01A, N02BA) | 2189 (6.7) | 2515 (6.9) |
| Opioids (N02A) | 62 (0.2) | 92 (0.3) |
| Psychotropic drugs^a^ | 821 (2.5) | 995 (2.7) |
| Triptans (N02CC) | 291 (0.9) | 251 (0.7) |
| Exposure status, n (%) |  |  |
| No use of paracetamol during pregnancy | 17 808 (54.1) | 20 157 (55.0) |
| Paracetamol use in one trimester | 8374 (25.4) | 9017 (24.6) |
| Paracetamol use in two trimesters | 4961 (15.1) | 5442 (14.9) |
| Paracetamol use in three trimesters | 1791 (5.4) | 2005 (5.5) |
| ^a^ Psychotropic drugs include: antidepressants (N06A), antipsychotics (N05A), antiepileptics (N03A), stimulants (N06BA), benzodiazepines (N05BA, N05CD), and benzodiazepine-like drugs (N05CF). | | |

**eTable 3.** Various patterns of paracetamol exposure during pregnancy in the 5-year cohort (n=32 934)

| **Exposure pattern** | | **N total=32 934,  n (%)** |
| --- | --- | --- |
| BY DURATION | Number of trimesters; |  |
|  | Use in one trimester | 8374 (25.4) |
|  | Use in two trimesters | 4961 (15.1) |
|  | Use in three trimesters | 1791 (5.4) |
|  | Number of 4-week intervals ; |  |
|  | 1-2 | 10 218 (31.0) |
|  | 3-4 | 3247 (9.9) |
|  | ≥5 | 1661 (5.0) |
|  | Days; |  |
|  | <28 days | 13 400 (40.7) |
|  | ≥28 days | 708 (2.2) |
|  |  |  |
| BY TIMING | Trimester; |  |
|  | Use in 1^st^ trimester | 8593 (26.1) |
|  | Use in 2^nd^/3^rd^ trimester^a^ | 6533 (26.8) |
|  | Gestational week; |  |
|  | GW 0-4 | 2338 (7.1) |
|  | GW 5-8 | 4042 (12.3) |
|  | GW 9-12 | 5851 (17.8) |
|  | GW 13-16 | 7416 (22.5) |
|  | GW 17-20 | 3241 (9.8) |
|  | GW 21-24 | 3589 (10.9) |
|  | GW 25-28 | 3986 (12.1) |
|  | GW 29+ | 2183 (6.6) |
|  | End of pregnancy | 2404 (7.3) |

^a^Use in 2^nd^ and / or 3^rd^ trimester, but not in 1^st^ trimester
GW, gestational week

**eTable 4.** Items composing the ASQ, CBCL, and EAS in MoBa Q-5year and sub-scale reliability information (Cronbach’s α)

|  | **Response**  **options** | **Items** | **Cronbach’s α** | |
| --- | --- | --- | --- | --- |
| ASQ |  |  |  |  |
| Communication skills | “Yes”  “A few times”  “Not Yet” | - Can tell at least two things about a common object^a^ - Can follow three directions that are unrelated to one another - Uses four- and five-word sentences - Uses -ed endings when talking about something that already happened - Uses comparison words (i.e., heavier, stronger, shorter) - Answers the questions “What do you do when you’re hungry?” and “What do you do when you’re tired?” correctly - Repeats sentences back correctly (“Jane hides her shoes for Mary to find”, “Al read the blue book under his bed”) | 0.65 |  |
| CBCL  Externalising behaviour |  |  |  |  |
| Attention problems | “Not true”  “Somewhat or somethimes true”  “Very true or often true” | - Cannot concentrate, cannot pay attention for long - Cannot sit still, restless, or overactive - Quickly shifts from one activity to another - Poorly coordinated or clumsy | 0.61 | 0.77 |
| Aggressive behaviour |  | - Cannot stand waiting, wants everything now - Defiant - Demands must be met immediately - Does not seem to feel guilty after misbehaving - Gets in many fights - Hits others - Punishment does not change his/her behaviour | 0.71 |  |
| CBCL  Internalising behaviour |  |  |  |  |
| Emotionality reactive | “Not true”  “Somewhat or somethimes true”  “Very true or often true” | - Disturbed by any change in routine | - | 0.61 |
| Anxious/depressed |  | - Clings to adult or too dependent - Gets too upset when separated from parents - Too fearful or anxious - Nervous, high strung, or tense - Unhappy, sad, or depressed - Self-conscious or easily embarrassed^b^ - Feelings are easily hurt^b^ | 0.61 |  |
| Somatic complaints |  | - Does not eat well - Stomach aches or cramps (without medical cause) - Vomiting, throwing up (without medical cause) | 0.22 |  |
| EAS |  |  |  |  |
| Emotionality | “Not at all typical”  “Not so typical”  “Neither/nor”  “Quite typical”  “Very typical” | - Cries easily - Gets upset or sad easily - Reacts intensely when upset | 0.75 |  |
| Activity |  | - Is always on the go - Is up and running as soon as he/she wakes up - Prefers quite inactive games to more active ones | 0.70 |  |
| Sociability |  | - Prefers playing with others rather than alone - Likes to be with people - Finds other people more fun than anything else | 0.71 |  |
| Shyness |  | - Is very sociable - Takes a long time to warm up to strangers - Very friendly with strangers | 0.70 |  |
| ^a^Item adapted from the 48-month ASQ. ^b^Items included only in the second version of Q-5year. | | | | |

**eTable 5.** Overview of the sources of the relevant variables in the MoBa and MBRN.

|  | **MoBa Q1**  **Pregnancy week 17-18** | **MoBa Q3**  **Pregnancy week 30** | **MoBa Q4**  **Six months after delivery** | **MoBa Q5-years**  **Child 5 years of age** | **MBRN Record** |
| --- | --- | --- | --- | --- | --- |
| Variables | - Medication use GW 0-13+ - Maternal health conditions - Education - Pre-pregnancy BMI - Folate intake - Smoking habits - Alcohol use - SCL-5 | - Medication use GW 13-29+ - Maternal health conditions - Smoking habits - Alcohol use - SCL-8 | - Medication use GW 30-delivery - Maternal health conditions - Smoking habits - Alcohol use | - ASQ - CBCL - EAS | - Maternal age at delivery - Marital status - Child sex - Birthweight - Gestational age - Malformations |
| MoBA, The Norwegian Mother and Child Cohort Study; MBRN, The Medical Birth Registry of Norway; SCL, Hopkins Symptoms Checklist (5 or 8-item version); ASQ, Ages and Stages Questionnaire; CBCL, Child Behaviour Checklist; EAS, Emotionality Activity and Shyness Temperament Questionnaire; GW, gestational week. | | | | | |

**eTable 6.** Balance of covariates between the exposed/unexposed groups

|  | **Standardised difference^a^** | | | | | | | | | |
| --- | --- | --- | --- | --- | --- | --- | --- | --- | --- | --- |
|  | **Model 1** | | **Model 2** | | **Model 3** | | **Model 4** | | **Model 5** | |
|  | Before weighting | After weighting | Before weighting | After weighting | Before weighting | After weighting | Before weighting | After weighting | Before weighting | After weighting |
| Maternal characteristics |  |  |  |  |  |  |  |  |  |  |
| Age at time of delivery | -0.071 | -0.006 | -0.054 | -0.027 | 0.029 | -0.023 | -0.030 | -0.020 | -0.063 | -0.005 |
| Married/cohabiting | -0.009 | 0.002 | -0.005 | 0.008 | -0.011 | 0.002 | -0.010 | -0.002 | -0.005 | 0.006 |
| Primiparous | 0.064 | -0.003 | 0.153 | -0.024 | 0.296 | -0.031 | 0.098 | -0.002 | 0.111 | -0.005 |
| University/college education | -0.017 | -0.003 | -0.025 | -0.011 | -0.009 | 0.011 | -0.000 | -0.012 | -0.031 | -0.008 |
| Pre-pregnancy BMI (kg/m^2^) | 0.116 | 0.002 | 0.197 | -0.003 | 0.319 | 0.028 | 0.138 | 0.004 | 0.153 | 0.002 |
| Folic acid supplement | 0.030 | 0.006 | 0.086 | 0.009 | 0.125 | -0.003 | 0.068 | -0.002 | 0.036 | 0.011 |
| Symptoms of anxiety/depression | 0.087 | 0.010 | 0.187 | 0.044 | 0.305 | 0.067 | 0.125 | 0.020 | 0.130 | 0.013 |
| Smoking during pregnancy |  |  |  |  |  |  |  |  |  |  |
| No | -0.082 | -0.018 | -0.085 | -0.032 | -0.088 | -0.000 | -0.054 | -0.022 | -0.091 | -0.020 |
| Yes | 0.066 | 0.044 | 0.084 | 0.069 | 0.079 | -0.019 | 0.054 | 0.034 | 0.072 | 0.051 |
| Stopped | 0.049 | -0.009 | 0.040 | -0.010 | 0.047 | 0.012 | 0.024 | 0.002 | 0.054 | -0.011 |
| Alcohol intake during pregnancy |  |  |  |  |  |  |  |  |  |  |
| No or minimal | -0.038 | -0.006 | -0.045 | -0.029 | -0.100 | 0.001 | -0.052 | -0.009 | -0.031 | -0.011 |
| Low to moderate | 0.036 | 0.011 | 0.050 | 0.040 | 0.088 | -0.004 | 0.052 | 0.015 | 0.030 | 0.015 |
| Frequent | 0.008 | -0.014 | -0.014 | -0.036 | 0.047 | 0.010 | 0.005 | -0.020 | 0.006 | -0.011 |
| Health conditions |  |  |  |  |  |  |  |  |  |  |
| Headache or migraine | 0.459 | -0.001 | 1.023 | 0.023 | 1.499 | 0.052 | 0.675 | 0.010 | 0.585 | 0.004 |
| Pain | 0.148 | 0.010 | 0.282 | 0.049 | 0.431 | 0.032 | 0.200 | 0.016 | 0.189 | 0.019 |
| Fever | 0.448 | -0.010 | 0.478 | 0.011 | 0.486 | 0.083 | 0.188 | 0.020 | 0.580 | -0.010 |
| Infections | 0.206 | 0.005 | 0.263 | 0.015 | 0.335 | 0.059 | 0.160 | 0.014 | 0.253 | 0.010 |
| Co-medications |  |  |  |  |  |  |  |  |  |  |
| NSAIDs | 0.193 | -0.004 | 0.289 | 0.018 | 0.463 | 0.028 | 0.245 | 0.011 | 0.204 | 0.003 |
| Opioids | 0.056 | -0.002 | 0.058 | -0.006 | 0.082 | -0.001 | 0.039 | 0.001 | 0.061 | -0.003 |
| Antidepressants | 0.021 | -0.001 | 0.058 | 0.004 | 0.112 | -0.003 | 0.036 | 0.003 | 0.043 | -0.002 |
| Antiepileptics | -0.001 | 0.001 | 0.003 | 0.010 | -0.004 | -0.035 | -0.007 | -0.002 | 0.007 | 0.005 |
| Antipsychotics | 0.003 | -0.001 | 0.009 | -0.013 | 0.016 | -0.005 | -0.016 | 0.001 | 0.025 | -0.005 |
| Stimulants | 0.023 | -0.002 | 0.018 | -0.000 | 0.017 | -0.001 | 0.008 | 0.002 | 0.025 | -0.001 |
| Benzodiazepines | 0.023 | 0.011 | 0.056 | 0.002 | 0.017 | 0.007 | 0.006 | 0.001 | 0.033 | 0.0025 |
| Benzodiazepine-like drugs | 0.034 | -0.003 | 0.068 | -0.008 | 0.101 | 0.004 | 0.041 | 0.003 | 0.055 | 0.002 |
| Triptans | 0.052 | -0.001 | 0.138 | 0.002 | 0.217 | 0.004 | 0.130 | -0.000 | 0.054 | 0.001 |
| Child characteristics |  |  |  |  |  |  |  |  |  |  |
| Boy | 0.012 | 0.001 | 0.022 | -0.012 | 0.034 | 0.014 | 0.018 | -0.004 | 0.013 | 0.002 |
| Interaction terms |  |  |  |  |  |  |  |  |  |  |
| Depression scores* headache/migraine | - | - | - | - | 0.232 | 0.004 | - | - | - | - |
| Pain conditions* headache/migraine | - | - | - | - | 1.219 | 0.040 | - | - | - | - |
| ^a^ Mean difference divided by the pooled standard deviation. Standardised differences <0.1 were considered acceptable. Model 1: Paracetamol use in one trimester vs. Never user. Model 2: Paracetamol use in two trimesters vs. Never user. Model 3: Paracetamol use in three trimesters vs. Never user.  Model 4: Paracetamol use in first trimester vs no use in first trimester  Model 5: Paracetamol use in second/third trimester and no use in first trimester vs no use during pregnancy | | | | | | | | | | |

**eTable 7.** Characteristics of generated stabilised weights

|  | **Estimated IPTW** | | **Estimated IPCW** | | **Estimated IPTW*IPCW** | |
| --- | --- | --- | --- | --- | --- | --- |
|  | Mean (SD) | Min-Max | Mean (SD) | Min-Max | Mean (SD) | Min-Max |
| Model 1 | 1.00 (0.38) | 0.33-9.81 | 1.00 (0.30) | 0.63-3.97 | 1.00 (0.50) | 0.27-10.91 |
| Model 2 | 1.00 (0.58) | 0.22-18.04 | 1.00 (0.31) | 0.64-3.78 | 1.00 (0.67) | 0.17-18.49 |
| Model 3 | 1.00 (0.58) | 0.09-19.34 | 1.00 (0.30) | 0.63-3.53 | 1.00 (0.68) | 0.07-22.53 |
| Model 4 | 1.00 (0.33) | 0.29-3.87 | 1.00 (0.30) | 0.63-3.40 | 1.00 (0.46) | 0.23-5.31 |
| Model 5 | 1.00 (0.47) | 0.27-14.16 | 1.00 (0.30) | 0.64-4.15 | 1.00 (0.59) | 0.24-16.47 |
| IPTW, inverse probability of treatment weight; IPCW, inverse probability of censoring weight, SD: standard deviation. Model 1: Paracetamol use in one trimester vs. Never user. Model 2: Paracetamol use in two trimesters vs. Never user. Model 3: Paracetamol use in three trimesters vs. Never user. Model 4: Paracetamol use in first trimester vs no use in first trimester  Model 5: Paracetamol use in second/third trimester and no use in first trimester vs no use during pregnancy | | | | | | |

**eTable 8.** Associations between exposure to paracetamol prior to pregnancy only and communication and behaviour problems in preschool-aged children

| **Communication and behavioural problems^a^** | **Total n** | **Patients with outcome, % of n** | **Unadjusted RR (95% CI)** | **Adjusted RR (95% CI)** |
| --- | --- | --- | --- | --- |
| Communication problems |  |  |  |  |
| Never user | 14 492 | 7.3 | Reference | Reference |
| Paracetamol use prior to pregnancy only | 2766 | 8.4 | 1.16 (1.01, 1.32) | 1.19 (1.02, 1.38) |
| Externalising problems |  |  |  |  |
| Never user | 14 454 | 9.3 | Reference | Reference |
| Paracetamol use prior to pregnancy only | 2770 | 9.6 | 1.03 (0.91, 1.16) | 0.99 (0.87, 1.14) |
| Internalising problems |  |  |  |  |
| Never user | 14 602 | 9.8 | Reference | Reference |
| Paracetamol use prior to pregnancy only | 2784 | 9.9 | 1.01 (0.90, 1.15) | 0.96 (0.84, 1.10) |
| RR, relative risk ^a^ Communication skills were assessed by the ASQ and behaviour problems by the CBCL. | | | | |

**eTable 9.** Associations between exposure to paracetamol prior to pregnancy only and temperamental traits in preschool-aged children

| **Temperament^a^** | **Total n** | **Mean T-score (SD)** | **Unadjusted β (95% CI)** | **Adjusted β (95% CI)** |
| --- | --- | --- | --- | --- |
| Emotionality |  |  |  |  |
| Never user | 14 573 | 49.6 (9.9) | Reference | Reference |
| Paracetamol use prior to pregnancy only | 2782 | 50.4 (10.0) | 0.75 (0.34, 1.15) | 0.36 (-0.08, 0.81) |
| Activity |  |  |  |  |
| Never user | 14 745 | 50.0 (10.1) | Reference | Reference |
| Paracetamol use prior to pregnancy only | 2807 | 49.1 (9.8) | -0.92 (-1.32, -0.52) | -0.80 (-1.23, -0.36) |
| Sociability |  |  |  |  |
| Never user | 14 736 | 49.9 (9.9) | Reference | Reference |
| Paracetamol use prior to pregnancy only | 2808 | 50.3 (9.8) | 0.35 (-0.05, 0.74) | 0.22 (-0.22, 0.66) |
| Shyness |  |  |  |  |
| Never user | 14 666 | 50.1 (10.0) | Reference | Reference |
| Paracetamol use prior to pregnancy only | 2785 | 50.3 (10.0) | 0.28 (-0.13, 0.69) | 0.35 (-0.10, 0.80) |
| ^a^ Measured by the EAS. | | | | |

**eTable 10.** Proportion of internalising behavioural problems among children born to mothers who used paracetamol in three trimesters and children born to mothers who did not use paracetamol according to percentiles of the propensity score

|  | **Exposed to paracetamol in three trimesters** | | | **Unexposed to paracetamol during pregnancy** | | |  |
| --- | --- | --- | --- | --- | --- | --- | --- |
| **Percentile^a^** | **Score^b^** | **Present for follow-up^c^,**  **n** | **Internalising behaviour,**  **% of n** | **Score^b^** | **Present for follow-up^c^,**  **n** | **Internalising behaviour,**  **% of n** | **Empirical RR^d^** |
| 0 - <1 | - | 0 | 0.0 | 0.004 | 151 | 10.6 | - |
| 1 - <5 | 0.006 | 4 | 50.0 | 0.006 | 801 | 7.2 | 7.03 |
| 5 - <10 | 0.008 | 7 | 28.6 | 0.008 | 960 | 10.7 | 2.78 |
| 10 - <25 | 0.011 | 29 | 3.4 | 0.011 | 2907 | 8.2 | 0.37 |
| 25 - <50 | 0.019 | 89 | 13.5 | 0.018 | 4705 | 9.1 | 1.50 |
| 50 - <75 | 0.053 | 201 | 11.4 | 0.042 | 4575 | 9.8 | 1.27 |
| 75 - <90 | 0.202 | 591 | 13.4 | 0.184 | 2319 | 12.8 | 1.06 |
| 90 - <95 | 0.341 | 300 | 12.7 | 0.338 | 589 | 12.4 | 1.01 |
| 95 - <99 | 0.523 | 411 | 10.5 | 0.508 | 381 | 12.1 | 0.85 |
| 99 – 100 | 0.753 | 122 | 18.0 | 0.732 | 58 | 12.1 | 1.54 |
| Overall | 0.310 | 1754 | 12.7 | 0.069 | 17 446 | 9.8 | 1.38 |
| RR, relative risk ^a^ Percentile of the propensity score for the entire population ^b^ Mean propensity score in percentile ^c^ Responded to CBCL internalising scale in Q5-years ^d^ Propensity-stratum-specific RR for internalising behaviour problem | | | | | | | |

**eTable 11.** Shyness temperament among children born to mothers who used paracetamol in two trimesters and children born to mothers who did not use paracetamol according to percentiles of the propensity score

|  | **Exposed to paracetamol in two trimesters** | | | **Unexposed to paracetamol during pregnancy** | | |  |
| --- | --- | --- | --- | --- | --- | --- | --- |
| **Percentile^a^** | **Score^b^** | **Present for follow-up^c^,**  **n** | **Mean T-score (SD)** | **Score^b^** | **Present for follow-up^c^,**  **n** | **Mean T-score (SD)** | **Empirical β^d^** |
| 0 - <1 | 0.042 | 5 | 44.1 (5.7) | 0.042 | 200 | 50.3 (10.4) | -6.03 |
| 1 - <5 | 0.052 | 29 | 47 (11.1) | 0.052 | 876 | 50.0 (9.8) | -3.07 |
| 5 - <10 | 0.059 | 56 | 53.5 (12.0) | 0.059 | 1053 | 50.2 (10.2) | 3.27 |
| 10 - <25 | 0.069 | 231 | 49.4 (9.6) | 0.069 | 3243 | 49.9 (10.1) | -0.50 |
| 25 - <50 | 0.090 | 443 | 49.1 (9.1) | 0.089 | 5208 | 50.0 (9.9) | -0.89 |
| 50 - <75 | 0.232 | 1237 | 49.6 (9.7) | 0.187 | 4307 | 50.1 (10.0) | -0.66 |
| 75 - <90 | 0.417 | 1523 | 49.8 (9.9) | 0.413 | 1810 | 50.6 (10.0) | -0.71 |
| 90 - <95 | 0.563 | 598 | 50.3 (9.7) | 0.559 | 440 | 50.0 (10.1) | 0.35 |
| 95 - <99 | 0.725 | 589 | 50.1 (10.3) | 0.722 | 320 | 49.8 (10.1) | 0.35 |
| 99 – 100 | 0.851 | 163 | 49.8 (10.4) | 0.844 | 55 | 50.4 (11.1) | 0.38 |
| Overall | 0.390 | 4874 | 49.9 (9.8) | 0.167 | 17 512 | 50.1 (10.0) | -0.48 |
| ^a^ Percentile of the propensity score for the entire population ^b^ Mean propensity score in percentile ^c^ Responded to EAS shyness in Q5-years ^d^ Propensity-stratum-specific β for the temperamental trait of shyness | | | | | | | |

**eTable 12.** Asymmetric trimming of the propensity score in analyses of the association between paracetamol exposure in three trimesters and internalising behaviour problems

|  | **Exposed to paracetamol in three trimesters** | | | **Unexposed to paracetamol during pregnancy** | |  |
| --- | --- | --- | --- | --- | --- | --- |
| **Trimming range** | **Present for follow-up, n** | **Internalising problems, % of n** | | **Present for follow-up, n** | **Internalising problems, % of n** | **Empirical RR** |
| Unrestricted | 1754 | | 12.7 | 17 446 | 9.8 | 1.38 |
| Restricted |  | |  |  |  |  |
| Common support | 1753 | | 12.7 | 17 248 | 9.8 | 1.38 |
| 1-99^a^ | 1734 | | 12.6 | 17 280 | 9.8 | 1.23 |
| Excluded | 20 | | 20.0 | 166 | 12.0 | 1.99 |
| 2.5-97.5^a^ | 1703 | | 12.6 | 17 019 | 9.8 | 1.29 |
| Excluded | 51 | | 13.7 | 427 | 11.9 | 1.39 |
| 5-95^a^ | 1651 | | 12.6 | 16 611 | 9.8 | 1.26 |
| Excluded | 103 | | 13.6 | 835 | 11.0 | 1.38 |
| 10-90^a^ | 1579 | | 12.5 | 15 750 | 9.6 | 1.26 |
| Excluded | 175 | | 13.7 | 1696 | 11.8 | 1.27 |
| RR, relative risk ^a^ Asymmetric trimming of the propensity score. | | | | | | |

**eTable 13.** Asymmetric trimming of the propensity score in analyses of the association between paracetamol exposure in two trimesters and shyness traits

|  | **Exposed to paracetamol in two trimesters** | **Unexposed to paracetamol during pregnancy** |  |
| --- | --- | --- | --- |
| **Trimming range** | **Present for follow-up, n** | **Present for follow-up, n** | **Empirical β** |
| Unrestricted | 4874 | 17 512 | -0.48 |
| Restricted |  |  |  |
| Common support | 4873 | 17 509 | -0.48 |
| 1-99^a^ | 4832 | 17 345 | -0.44 |
| Excluded | 42 | 167 | -1.80 |
| 2.5-97.5^a^ | 4755 | 17 091 | -0.55 |
| Excluded | 119 | 421 | 0.21 |
| 5-95^a^ | 4615 | 16 698 | -0.66 |
| Excluded | 259 | 814 | 0.39 |
| 10-90^a^ | 4359 | 15 863 | -0.53 |
| Excluded | 515 | 1649 | -0.34 |
| ^a^ Asymmetric trimming of the propensity score. | | |  |
|  | | | |

**eTable 14.** Principal component analysis (PCA)

|  | **Variable** | **Component 1** | **Component 2** | **Unexplained** |
| --- | --- | --- | --- | --- |
| ASQ | Communication | 0.2080 | -0.0870 | 0.8675 |
| CBCL Externalising | Attention | 0.4559 | 0.2065 | 0.4255 |
|  | Aggression | 0.4853 | 0.0808 | 0.3966 |
| CBCL Internalising | Emotionally reactive | 0.3460 | -0.1660 | 0.6188 |
|  | Anxious/depressed | 0.3450 | -0.2683 | 0.5271 |
|  | Somatic complaints | 0.2516 | -0.0644 | 0.8228 |
| EAS | Emotionality | 0.4032 | -0.0551 | 0.5661 |
|  | Activity | 0.2096 | 0.4773 | 0.5199 |
|  | Sociability | 0.0198 | 0.5391 | 0.4840 |
|  | Shyness | 0.0666 | -0.5640 | 0.4052 |
| ASQ, Ages and Stages Questionnaire; CBCL, Child Behaviour Checklist; EAS, Emotionality Activity and Shyness Temperament Questionnaire. | | | | |

**References**

1. Squires J, Bricker D, Potter L. The ASQ Users's Guide. Second ed. Baltimore: Paul H. Brookes Publishing Co.; 1999.

2. Achenbach TM, Ruffle TM. The Child Behavior Checklist and related forms for assessing behavioral/emotional problems and competencies. *Pediatrics in Review.* 2000;21:265-271.

3. Novik TS. Validity of the Child Behaviour Checklist in a Norwegian sample. *European Child and Adolescent Psychiatry.* 1999;8:247-254.

4. Koot HM, Van Den Oord EJ, Verhulst FC, Boomsma DI. Behavioral and emotional problems in young preschoolers: cross-cultural testing of the validity of the Child Behavior Checklist/2-3. *Journal of Abnormal Child Psychology.* 1997;25:183-196.

5. Kristensen S, Henriksen TB, Bilenberg N. The Child Behavior Checklist for Ages 1.5-5 (CBCL/1(1/2)-5): assessment and analysis of parent- and caregiver-reported problems in a population-based sample of Danish preschool children. *Nordic Journal of Psychiatry.* 2010;64:203-209.

6. Tambs K, Røysamb E. Selection of questions to short-form versions of original psychometric instruments in MoBa. *Norsk Epidemiologi.* 2014;24:195-201.

7. Strand BH, Dalgard OS, Tambs K, Rognerud M. Measuring the mental health status of the Norwegian population: a comparison of the instruments SCL-25, SCL-10, SCL-5 and MHI-5 (SF-36). *Nordic Journal of Psychiatry.* 2003;57:113-118.

8. Stürmer T, Rothman KJ, Avorn J, Glynn RJ. Treatment effects in the presence of unmeasured confounding: dealing with observations in the tails of the propensity score distribution-a simulation study. *American Journal of Epidemiology.* 2010;172:843-854.

9. van Gelder M, Vorstenbosch S, Te Winkel B, van Puijenbroek EP, Roeleveld N. Using web-based questionnaires to assess medication use during pregnancy: a validation study in 2 prospectively enrolled cohorts. *American Journal of Epidemiology.* 2018;187:326-336.

10. Skurtveit S, Selmer R, Odsbu I, Handal M. Self-reported data on medicine use in the Norwegian Mother and Child cohort study compared to data from the Norwegian Prescription Database. *Norsk Epidemiologi.* 2014;24:209-216.

11. Kaiser HF. The varimax criterion for analytic rotation in factor analysis. *Psychometrika.* 1958;23:187-200.

12. Clark LA. Temperament as a unifying basis for personality and psychopathology. *Journal of Abnormal Psychology.* 2005;114:505-521.
